# Supplementary material for: WordCommentsAnalyzer: A windows software tool for qualitative research
Source: F1000Res. 2018 Sep 4;7:536. Originally published 2018 May 3. [Version 2] doi: 10.12688/f1000research.14819.2 (PMC6137411; doi:10.12688/f1000research.14819.2)
Supplement: Supplementary file 3 [file f1000research-7-17614-s0002.tgz › b2a79496-4ada-47c4-9682-a99ce867836d.zip!/Eight Iranian Health Journals/Salmand_Iranian Journal of Ageing _43.docx]

© Salmand_Iranian Journal of Ageing . This work is licensed under CC BY-NC 4.0 (https://creativecommons.org/licenses/by-nc/4.0/).

Volume & Issue: دوره 2 - شماره 3

Title: جمعیت‌ شناسی سالمندان در ایران براساس سرشماری‌های 1325 تا 1385

http://salmandj.uswr.ac.ir/article-1-43-fa.html

اهداف: یکی از مسائل عمده جمعیتی عمدتا در کشورهای جهان مسئله سال‌خوردگی جمعیت و پیامدهای ناشی از آن است. هدف این مقاله بررسی سطح و روند جمعیت 60 سال و بالاتر ایران، نسبت جنسی سالمندان، رشد جمعیت سالمند، شاخص سالمندی و نسبت وابستگی است.  مواد و روش‌ها: در حال حاضر کشور ایران نیز مرحله انتقال ساختار سنی جمعیت از جوانی به سال‌خوردگی را تجربه می‌کند. با اینکه جمعیت سالمند سهم کمی از جمعیت کشور را به خود اختصاص داده است اما فزونی سرعت رشد جمعیت سالمند در مقایسه با رشد جمعیت کل کشور و پیش‌بینی افزایش تعداد و سهم سال‌خوردگان (جمعیت 60 سال و بالاتر) در سال‌های آتی لزوم برنامه‌ریزی آینده‌نگر برای کنترل مسائل مربوط به این گروه از جمعیت را مورد تأکید قرار می‌دهد. برای دستیابی به این هدف به مطالعه اسنادی داده های سرشماری عمومی نفوس و مسکن مرکز آمار ایران طی سالهای 85-1335 پرداخته شده است. یافته‌ها: شاخصهای مهم جمعیتی همچون نسبت سالمندی، نسبت جنسی سالمندان، رشد جمعیت سالمندان، شاخص سالمندی و نسبت وابستگی جمعیت سالمندان در ایران برای سال 1385 به ترتیب برابر 7/3، 108، 2/5، 29 و 43/4 برآورد شده است. نتیجه‌گیری: با توجه به آهنگ پرشتاب کاهش باروری ایران طی دو دهه اخیر، پیش بینی می شود حرکت به سمت سالخوردگی جمعیت تسریع شود و با استناد به روندهای مشاهده شده باروری و مرگ و میر و نگاهی به محتمل ترین مسیرهای تغییر آنها در آینده برآورد می شود که شروع قرن پانزدهم هجری شمسی سرآغاز روند روبه افزایش جمعیت سالخورده در ایران باشد.

Title: بررسی ارتباط رفتارهای ارتقادهنده سلامت با سطح فعالیت‌ های روزمره زندگی و فعالیت‌ های زندگی با ابزار در سالمندان غرب تهران 1386

http://salmandj.uswr.ac.ir/article-1-44-fa.html

اهداف: همان‌طور که طول عمر افراد افزایش می‌یابد، اهمیت رفتارهای ارتقادهنده سلامت نیز، با توجه به حفظ کارکرد و استقلال افراد و افزایش کیفیت زندگی آن‌ها، روز به روز بیشتر آشکار می‌شود. پژوهش حاضر با هدف تعیین ارتباط رفتارهای ارتقادهنده سلامت با سطح ADL و IADL در سالمندان غرب تهران انجام گرفته است. مواد و روش‌ها: این مطالعه از نوع همبستگی بود که بر روی 410 نفر از افراد بالای 60 سال ساکن منطقه غرب تهران انجام گرفت. روش نمونه‌گیری از نوع چند مرحله‌ای بود و از ابزار پژوهش شامل چک‌لیست مربوط به رفتارهای ارتقادهنده سلامت و پرسش‌نامه مربوط به فعالیت‌های جسمی، که از دو بخش سوالات مربوط به ADL (فعالیت‌های روزمره زندگی) و IADL (فعالیت‌های ابزاری زندگی) تشکیل شده است، استفاده شد. اطلاعات مربوط به واحدهای مورد پژوهش از طریق مصاحبه گردآوری شد. برای تحلیل داده‌ها از آمار توصیفی و آزمون تی- تست استفاده شد. یافته‌ها: بین فعالیت‌های روزمره زندگی (ADL) و رفتارهای «انجام ورزش یا پیاده روی»، «مصرف شیر، لبنیات و غذاهای گوشتی»، «مصرف میوه و سبزیجات تازه»، «مصرف غذای کم‌نمک» و «مصرف غذای کم‌چرب» ارتباط معنی‌داری وجود داشت (P<0.05). همچنین بین فعالیت‌های ابزاری زندگی (IADL) و رفتارهای «نکشیدن سیگار»، «مصرف‌نکردن الکل»، «انجام ورزش یا پیاده‌روی»، «مصرف غذای کم‌نمک»، «مصرف غذای کم‌چرب»، «مصرف شیر، لبنیات و غذاهای گوشتی» و «مصرف میوه و سبزیجات تازه» ارتباط معنی‌داری وجود داشت (P<0.05). نتیجه‌گیری: مطالعه نشان داد رفتارهای ارتقادهنده سلامت با ADL و IADL ارتباط معنی‌داری داشت. لذا مراقبت دهنده‌های بهداشتی باید زمینه‌های تسهیل رفتارهای ارتقادهنده سلامت را از طریق برنامه های سلامتی مثل رژیم غذایی صحیح، ورزش منظم و بررسی دوره‌ای وضعیت جسمانی فراهم آورند تا موجب افزایش فعالیت‌های جسمی در سالمندان شده و از این طریق باعث حفظ و ارتقا زندگی سالم و فعال برای آن‌ها شوند.

Title: وضعیت تغذیه سالمندان در اصفهان (سال 1386)

http://salmandj.uswr.ac.ir/article-1-45-fa.html

اهداف: هدف از مطالعه حاضر، مشخص کردن وضعیت تغذیه در سالمندان و عوامل موثر بر آن می باشد. مواد و روش‌ها: در این مطالعه مقطعی 248 سالمند به‌طور تصادفی انتخاب شدند و وضعیت تغذیه آن‌ها توسط پرسش‌نامه MNA (Mini Nutritional= Assessment) که برای سالمندان ایران استاندارد شده، مورد ارزیابی قرار گرفت. برای سنجش ارتباط بین وضعیت تغذیه و فاکتورهای دموگرافیک از مدل رگرسیون لجستیک استفاده شد. یافته‌ها: 40درصد سالمندان وضعیت تغذیه‌ای نامناسب داشتند. در 67.5درصد آن‌ها BMI بالای 25 بود. در بین عوامل دموگرافیک، رابطه سوءتغذیه با سطح تحصیلات CI95%=0.23-0.77) و (OR=0.4، درآمد بالا CI95%=1.5-7.5) و (OR=3.4 و چاقی CI95%=1.6-31) و (OR=7 از نظر آماری معنی‌دار بود. نتیجه‌گیری: با توجه به شیوع نسبتا بالای اختلال تغذیه در سالمندان، و با عنایت به اینکه امکان مداخله مناسب در اغلب عوامل موثر بر آن وجود دارد، اهمیت توجه به اصلاح تامین معاش سالمندان و تدارک برنامه‌های مناسب تغذیه‌ای و ورزشی و همچنین کنترل وزن آن‌ها بیشتر روشن می‌شود.

Title: سوءمصرف دارو و حلال‌های شیمیایی در سالمندان اهوازی

http://salmandj.uswr.ac.ir/article-1-46-fa.html

اهداف: پژوهش‌های فراوانی به بروز و شیوع وابستگی‌های شیمیایی دارویی جدید اشاره کرده اند. سوءمصرف حلال‌های شیمیایی دارویی یکی ازاین سوء مصرف‌ها است. این بررسی به مطالعه وابستگی و سوء‌مصرف مواد شیمیایی در سالمندان اهوازی در سال 1386 می‌پردازد. مواد و روش‌ها: پژوهش با روش توصیفی – اکتشافی و با تکنیک‌های پرسش‌نامه، مصاحبه تشخیصی - بالینی و مطالعه ِ اسنادی پرونده‌های پزشکی و گزارش‌های بالینی ـ بیماران داوطلب اجرا گردید. جامعه آماری شامل سالمندان یکی از مناطق شهری اهواز است که به صورت نمونه‌گیری تصادفی در مجموع 74 پرونده پزشکی مطالعه شده و با 30 نفر از سالمندان داوطلب مصاحبه گردید. درنهایت نیز آزمون وابستگی دارویی سالمندان(EDAQ (در مورد آن‌ها اجرا شد. یافته‌ها: X2 نشان داد بین متغیرهای سن و سابقه مصرف مواد مخدر با یافته‌ها معناداری فرضیه‌ها با آزمون سوءمصرف مواد دارویی و شیمیایی رابطه معناداری وجود دارد؛ به‌طوری‌که بین سوء مصرف حلال‌هایی مثل چسب و بنزن‌ها با سابقه سوءمصرف مشتقات درجه دوم تریاک مثل مرفین، تبائین و هروئین رابطه معنادار، قوی‌تر بود. مصاحبه‌های بالینی ـ تشخیصی معین کرد وابستگی روانی ایجادشده توسط این مشتقات نقش مهمی در این نوع سوء مصرف به حلال‌ها دارد. نوع مصرف سالمندان بیشتر استنشاقی در ادامه تجویز دارویی پزشک معالج است. نتیجه‌گیری: هرچند شیوع‌شناسی این عارضه در پرونده‌های نمونه نشانگر درصد متوسط به پایین در این وابستگی شیمیایی است، اما در مجموع این پدیده قابل مطالعه و بررسی است. در پایان پژوهشگران به اجتناب از این نوع سوءمصرف جدید نظر داده و به نظارت بر رفتارها و گستره کنش متقابل این گونه رفتارهای اعتیادآور اشاره نموده اند. گزینه بهتر اینکه در توزیع داروها و مواد و حلال‌های شیمیایی و تجویز آن‌ها از سوی پزشک سالمند دقت بیشتری صورت پذیرد.

Title: تأثیر آرام‌سازی پیش‌رونده عضلانی بر پرفشاری خون سالمندان مبتلا به سکته قلبی

http://salmandj.uswr.ac.ir/article-1-47-fa.html

اهداف: پرفشاری خون سومین عامل مرگ و میر در جهان بوده و هزینه درمانی بالایی دارد. استفاده از روش‌های درمانی غیردارویی همانند آرام‌سازی پیش‌رونده عضلانی می‌تواند باعث کاهش فشارخون و عوارض خطرناک ناشی از آن مثل سکته قلبی شود. از این‌رو پژوهش حاضر به منظور تعیین تاثیر آرام‌سازی پیش‌رونده عضلانی بر پرفشاری خون بیماران سکته قلبی انجام شد. مواد و روش‌ها: این مطالعه نیمه‌تجربی (طرح گروه کنترل نامعادل) در سال 86 بر روی 40 بیمار مبتلا به سکته قلبی با سابقه پرفشاری خون اولیه در بیمارستان امیراعلم تهران انجام شد. بیماران به‌صورت تصادفی به دو گروه مداخله و کنترل تقسیم شدند و قبل از انجام مداخله اطلاعات دموگرافیک و علایم حیاتی ثبت گردید. در گروه مداخله روزانه 20 جلسه به‌مدت 3 روز تکنیک آرام‌سازی پیش‌رونده عضلانی انجام و پس از اتمام هر جلسه مجددا فشار خون بیماران کنترل و ثبت گردید. در گروه کنترل بدون انجام مداخله پس از 20 دقیقه مجددا فشار خون کنترل و ثبت گردید و نتایج با استفاده از آزمون‌های آماری مورد تجزیه و تحلیل قرار گرفت. یافته‌ها: میانگین سنی گروه مداخله 5/7±60/45 و در گروه کنترل 5/9±62/35 بود. نتایج نشان داد که میانگین فشارخون سیستولیک و دیاستولیک بعد از مداخله کاهش یافته و گروه کنترل معنی دار بوده (P<0/001) و همچنین میانگین اختلاف فشار خون سیستولیک و دیاستولیک قبل و بعد از مداخله نسبت به گروه کنترل کاملا معنی دار بود (P<0/001). نتیجه‌گیری: آرام‌سازی پیش‌رونده عضلانی باعث کاهش فشارخون سیستولیک و دیاستولیک در افراد مبتلا به سکته قلبی با سابقه پرفشاری خون می شود و می‌تواند به عنوان درمان کمکی در کنار درمان دارویی استفاده شود.

Title: بررسی تأثیر سن بر عملکرد شنوایی و سیر پیرگوشی در مراکز ارزیابی شنوایی شهر تهران

http://salmandj.uswr.ac.ir/article-1-48-fa.html

اهداف: کم‌شنوایی یکی از مشکلات شایع سلامتی است که در سنین بالا شیوع بیشتری دارد. مطالعه حاضر با هدف برسی تاثیر سن بر آستانه‌های شنوایی و توانایی بازشناسی واژه روی گروهی از افراد در محدوده سنی 30 تا 100 سال صورت گرفت. مواد و روش‌ها: مطالعه حاضر از اردیبهشت ماه 1384 تا مهرماه 1386 روی 190 نفر (%53.68 مرد و %46.32 زن) در هفت دهه سنی در شهر تهران انجام شد. این افراد کسانی بودند که به دلیل احساس وجود نوعی مشکل شنوایی، برای بررسی وضعیت شنوایی خود مراجعه کرده بودند. از بین مراجعان، افراد بدون سابقه آسیب شنوایی و قرارگرفتن در معرض عوامل ایجاد کننده کم‌شنوایی مورد بررسی قرار گرفته؛ و ادیومتری تون- خالص، ارزیابی توانایی بازشناسی گفتار، و ادیومتری امپدانس برای آن‌ها انجام شد. یافته‌ها: بین ثبت رفلکس‌های صوتی با سن و همچنین با میانگین کم‌شنوایی، ارتباط معکوس معناداری وجود داشت. کاهش حساسیت شنوایی بین دهه‌های سنی مورد مطالعه از لحاظ آماری معنادار بود؛ و در تمام فرکانس‌های مورد بررسی، میزان کم‌شنوایی در مردان نسبت به زنان بالاتر بود و اختلاف آن‌ها در فرکانس های بالا معنادار به دست آمد. بین گروه‌های سنی مورد مطالعه در امتیاز بازشناسی واژه نیز تفاوت معناداری مشاهده شد. وزوز دائمی در 12/63درصد افراد، سرگیجه/ گیجی در 6/84درصد، و سابقه استفاده از سمعک در 4/21درصد افراد گزارش گردید. نتیجه‌گیری: حساسیت شنوایی به تدریج و به طور پیش‌رونده با افزایش سن، کاهش می‌یابد. با توجه به تاثیر کم‌شنوایی و برخی اختلالات همراه آن به ویژه وزوز و سرگیجه/ گیجی بر توانایی ارتباطی و کیفیت زندگی افراد، و شیوع بالاتر آن‌ها نسبت به سایر اختلالات در سالمندان، برنامه‌ریزی‌های علمی و اجرایی جهت شناسایی و درمان مشکلات شنوایی در این جمعیت ضرورت دارد.

Title: بررسی رابطه سن با میزان گلبول‌های سفید خون محیطی در بیماران مبتلا به عفونت منتشر در دو بیمارستان تهران

http://salmandj.uswr.ac.ir/article-1-49-fa.html

اهداف: مقدار طبیعی WBC در افراد مسن مختصری کاهش یافته ولی در Sepsis میزان آن افزایش می یابد. لکوپنی و لکوسیتوز به عنوان ریسک فاکتورهای همراه با باکتریمی در مورتالیتی افراد مسن نقش دارد. این تحقیق جهت شناخت رابطه سن با تغییرات WBC بیماران مبتلا به Sepsis انجام گرفت. مواد و روش‌ها: روش تحقیق مورد شاهدی بوده که روی بیماران مبتلا به Sepsis در بیمارستان های لقمان حکیم و بوعلی تهران در سال های 1385-1380 انجام گرفت. یافته‌ها: 130 بیمار در دو گروه 65 ساله و بالاتر (مورد) و 64 ساله و جوانتر (شاهد) بررسی شدند. میانگین گلبول سفید خون بیماران گروه مورد 1424/2±17061/54 و در گروه شاهد 9888±13567/7 بوده که بین میانگین WBC در دو گروه اختلاف معنی‌دار آماری مشاهده نشد. ولی در میانگین تعداد نبض و درجه حرارت دهانی بیماران مسن و جوان اختلاف معنی دار بود (p<0/05) در بیماران مسن بین سن با سابقه عفونت اخیر و نیز سابقه بستری در یک ماه گذشته و همچنین بین منشا احتمالی عفونت و WBC رابطه معنی دار آماری وجود داشت (p<0/05). در هیچ یک از دو گروه بین مرگ با WBC و نیز مرگ با منشا احتمالی عفونت رابطه معنی دار وجود نداشت. نتیجه‌گیری: بین WBC و سن در بیماران مسن رابطه معنی دار آماری مشاهده نشد. Sepsis در افراد مسن می تواند بدون تب و تعداد نبض در دقیقه کمتری تظاهر نموده و سابقه عفونت و همچنین سابقه بستری در بیمارستان در یک ماه اخیر در ارزیابی این بیماران اهمیت دارد.

Title: ارزیابی میزان اثربخشی و هزینه در مقابل سود واکسیناسیون آنفلوانزا در سالمندان آسایشگاه خیریه کهریزک

http://salmandj.uswr.ac.ir/article-1-50-fa.html

اهداف: تغییرات دستگاه ایمنی در سالمندان موجب می‌شود که ابتلا به آنفولانزا با شدت و عوارض بیشتری در سالمندان همراه باشد. چنانچه بیماری آنفلوانزا یکی از علل اصلی مرگ‌ومیر در سالمندان است. همچنین شیوع آنفلوانزا در خانه‌های سالمندان به مراتب بیشتر است. هدف از این مطالعه بررسی تاثیر واکسن آنفلوانزا بر میزان وقوع عفونت آنفلوانزا و نیز مقایسه هزینه‌هایی که برای دارودرمانی، آزمایشات و رادیولوژی در دو گروه تحت واکسیناسیون و گروه کنترل صرف شده است، می‌باشد. این مطالعه در ماه‌های شیوع آنفلوانزا از آذر ماه سال 1384 تا شهریور 1385 در آسایشگاه خیریه کهریزک انجام گردیده است. مواد و روش‌ها: این یک مطالعه شاهد موردی آینده‌نگر بوده که روی 941 نفر از سالمندان مقیم آسایشگاه خیریه کهریزک انجام شده است. در این مطالعه واکسیناسیون بر روی 389 نفر از افراد 60 ساله و بالاتر انجام گرفته است. در مقابل گروه کنترل، شامل 557 نفر بوده‌اند که واکسن دریافت نکردند. در دو گروه میزان بروز بیماری شبه‌آنفلوانزا، هزینه‌های آزمایشگاه، دارودرمانی، رادیولوژی و نیز میزان کل مرگ و میر در دو گروه در مدت 10 ماه ثبت گردید. یافته‌ها: 10/9درصد از افراد مورد مطالعه مبتلا به بیماری شبه‌آنفلوانزا گردیدند، این نسبت در گروه واکسینه‌شده 7/2درصد و در گروه غیرواکسینه 13/4درصد بود. این اختلاف ازنظر آماری با 0/05P< معنادار بود. ازنظر میانگین هزینه‌های دارویی، اختلاف در دو گروه مشاهده شد (4918±1174 ریال درگروه واکسینه درمقابل 8710±2521 ریال) که با 0/01P< معنادار بود. اختلاف میانگین هزینه‌های آزمایشگاهی دو گروه با 0/0001P< معنادار بود. ( 408479±179494 ریال در گروه غیرواکسینه درمقابل 119362±48500 ریال در گروه واکسینه). میانگین هزینه‌های کل ( هزینه رادیولوژی، آزمایشگاه و داروها و واکسن) در گروه واکسینه 119962±90690 ریال و در گروه غیرواکسینه 413634±183015 ریال که اختلاف آن‌ها ازنظر آماری با 0/0001P< معنادار بود. میزان کل مرگ‌ومیر در گروه واکسینه 14/9درصد درحالی‌که در گروه غیرواکسینه 11/8درصد بود که ازنظر آماری اختلاف بین دو گروه معنادار نبود. بعد از تصحیح ازنظر سن، جنس و بیماری‌های زمینه‌ای 1/04Odds Ratio= شد، که بیانگر عدم تأثیر واکسیناسیون در میزان کل مرگ‌ومیر بود. نتیجه‌گیری: این مطالعه نشان داد که واکسیناسیون آنفلوانزا به‌طور مشخصی میزان وقوع بیماری و هزینه‌های مربوط به درمان را در گروه واکسینه نسبت‌به گروه غیرواکسینه کاهش می‌دهد. باتوجه‌به شیوع بیشتر آنفلوانزا در خانه‌های سالمندان، این مطالعه اثربخشی این واکسن درسالمندان بستری در خانه‌های سالمندان را نشان می‌دهد.
